# The risk of foot-and-mouth disease becoming endemic in a wildlife host is driven by spatial extent rather than density

**DOI:** 10.1371/journal.pone.0218898

**Published:** 2019-06-26

**Authors:** Simon Croft, James N. Aegerter, Giovanna Massei, Graham C. Smith

**Affiliations:** National Wildlife Management Centre, Animal and Plant Health Agency, Sand Hutton, York, United Kingdom; Panstwowy Instytut Weterynaryjny - Panstwowy Instytut Badawczy w Pulawach, POLAND

## Abstract

In the past 20 years, free living populations of feral wild boar have re-established in several locations across the UK. One of the largest populations is in the Forest of Dean where numbers have been steadily increasing since monitoring began in 2008, with estimates from 2016 reporting a population of more than 1500. Feral wild boar have significant ecological and environmental impacts and may present a serious epidemiological risk to neighbouring livestock as they are a vector for a number of important livestock diseases. This includes foot-and-mouth disease (FMD) which is currently absent from the UK. We developed an individual-based spatially explicit modelling approach to simulate feral wild boar populations in the Forest of Dean (England, UK) and use it to explore whether current or future populations might be sufficient to produce long-lived outbreaks of FMD in this potential wildlife reservoir. Our findings suggest that if you exclude the spread from feral wild boar to other susceptible species, the current population of boar is insufficient to maintain FMD, with 95% of unmanaged simulations indicating disease burn-out within a year (not involving boar management specifically for disease). However, if boar are allowed to spread beyond their current range into the adjacent landscape, they might maintain a self-sustaining reservoir of infection for the disease.

## Introduction

The abundance and distribution of feral wild boar are growing worldwide, together with the economic and environmental impact they produce [1–3]. In the UK, wild boar were hunted to extinction 300 years ago but have re-established notable free-living populations in the south-east of England (Kent and Sussex), in the Forest of Dean (Gloucestershire) and in parts of Scotland [4] as a result of escapes or deliberate releases. Founding populations remained initially small, seemingly controlled by *ad-hoc* hunting. In the UK only the land-owner’s permission is required to shoot boar on private land and no records of hunting effort are collected. However, in the Forest of Dean, numbers of feral wild boar have steadily increased since 2013, when regular censuses started on Forestry Commission (FC) owned land; growing from circa 500 animals (2013) to 1562 (2016) (95% confidence interval ranging from 1095 to 2296) [5]. Despite a program of culling across the FC estate (with annual targets to achieve a specified management objective), there remains concern that this population may grow and spread further, as feral wild boar generally show rapid population growth due to the early onset of sexual maturity, large litters, their potential to produce more than one litter a year [6] as well as their ability to disperse large distances [4]. This might produce more substantial and widespread direct impacts on landscapes which include economic damage to crops and woodland [2], nuisance to residents and users of affected landscapes (damage to gardens and recreational areas, raiding garbage, intimidating dog-walkers) as well as being vectors for a variety of serious livestock diseases including foot-and-mouth disease (FMD) [7, 8] and African and classical swine fever.

Foot-and-mouth disease is a highly infectious viral disease, primarily associated with artiodactyls (even-toed ungulates), that can produce severe epidemics in susceptible livestock along with significant economic consequences to international trade [9]. Potential impacts of FMD on the UK economy mean that prevention is a key priority for the Department for Environment, Food and Rural Affairs (Defra) who conduct regular risk assessment and contingency exercises to establish a robust outbreak response. The establishment and recent increase in abundance of feral wild boar in the Forest of Dean therefore requires work to quantify the disease risk it poses [10].

Individual- or agent- based models (IBMs) have become a popular tool in ecology and in particular to explore scenarios involving wildlife management and epidemiological risk [11, 12] with several existing studies focusing specifically on the simulation of wild boar populations either across theoretical landscapes [13] or simulations of real landscapes [14, 15]. These process driven models seek to represent biologically realistic systems at low level (individuals or single sounders of boar) and provide a platform to directly incorporate key biological or ecological processes, individual behaviours [16] or explore *in-silico* the consequences of environmental change or management options [17]. Key advantages of IBMs are that, unlike more traditional population level analytical models [14], model accuracy remains consistent even at small population sizes, capturing phenomena such as stochastic die-out which is particularly important for spatial models and understanding disease persistence and eradication. In addition, the smaller geographical grain at which these models can be applied permit a more nuanced and informative description of real-world landscapes and their heterogeneity.

Epidemiological modelling using ecological data from the Sussex population [14] and disease transmission data from Pakistan indicated that classical swine fever (CSF) could be sustained in small populations similar to that now seen in the Forest of Dean [4]. This prompts the requirement to re-assess the risks of disease establishment and maintenance for important diseases such as FMD, as well as exploring the principles of disease prevention or disease outbreak response.

Here, we outline a novel individual-based model incorporating a flexible spatial framework and epidemiological components to simulate the spread of feral wild boar and FMD across a real-world landscape. We then use this model to assess the risks posed by an FMD outbreak in the existing Forest of Dean boar population, thought to be the largest in the UK, and predict how disease risks may change under various management scenarios. Our principle question is to identify whether, or under what conditions, FMD becomes endemic in this wildlife reservoir (i.e. self-sustaining within the feral wild boar), how long outbreaks of FMD might persist if the virus does not become endemic, and to help inform the design of prophylactic management strategies to mitigate this risk.

## Methods

### Study site

**Fig 1:**
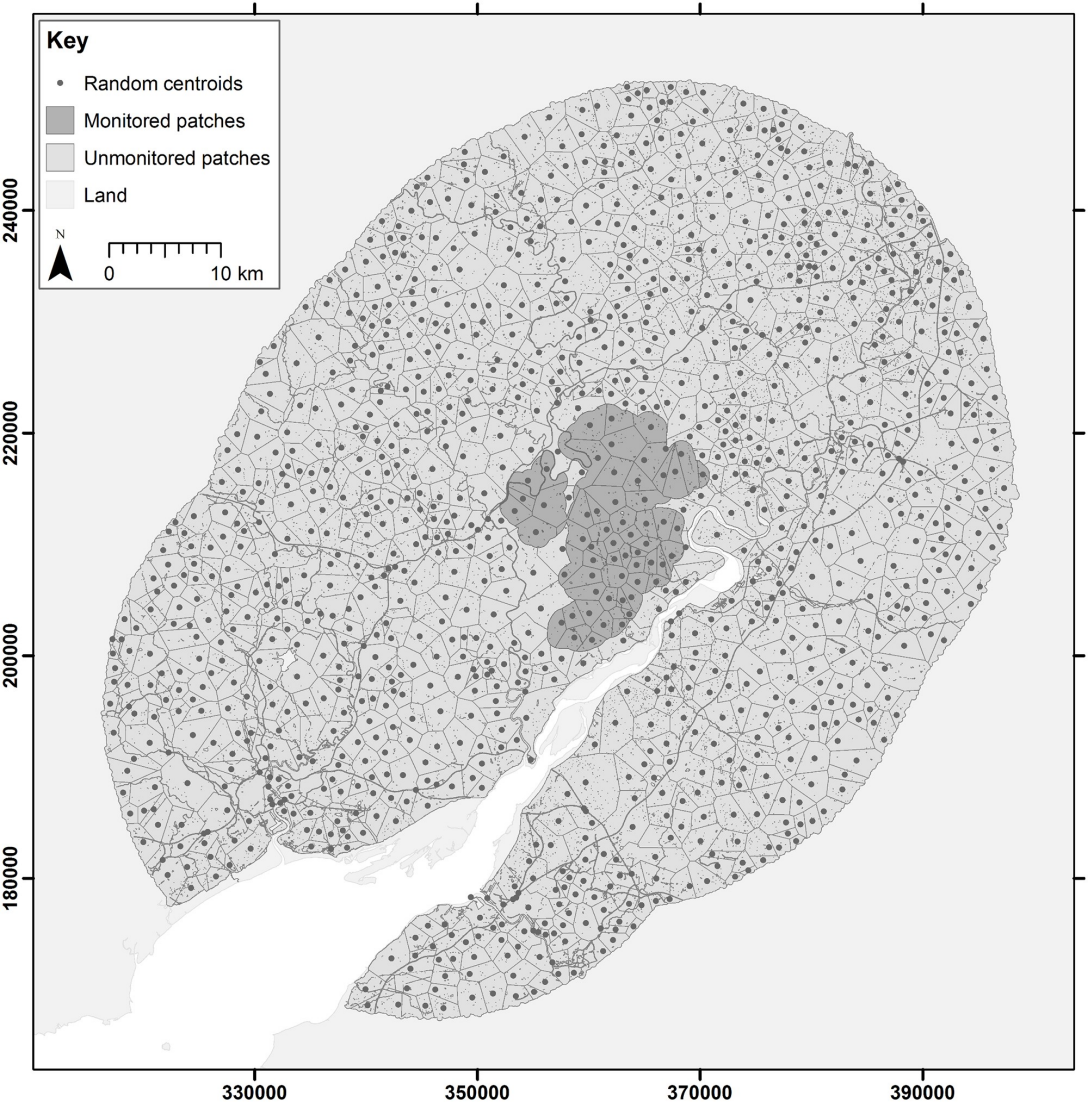
Map of study extent around the Forest of Dean. Model considers a 25 km buffer around the forest estate separated into two regions: that currently monitored (shaded) and that unmonitored (unshaded). Each of these regions are divided into randomised patches of approximately 4 km^2^.

The Forest of Dean (e.g. 51.80°N, 02.52°W) is a richly wooded landscape running along the western bank of the River Severn in south-western England. It includes an extensively forested core area owned by the Forestry Commission (hereafter the FC estate) comprising a fragmented 75 km^2^ of coniferous, deciduous and mixed stands, as well as a wider landscape rich in pasture and smaller woodlands largely in private ownership (Fig 1). Our study extent describes a 25 km buffer around the FC estate in the Forest of Dean.

Since their detection in 2008, the population of wild boar has been monitored across the FC estate with large scale systematic surveys and managed using culls (restricted to the FC estate). The current population appears largely limited to the core area hosting 1562 individuals in 2016, at a density of 20.9/km^2^ [5]. Despite a cull of 422 boar in 2015 this represents an increase in population size of approximately 43% from 2015, although accounting for differences in survey timing this is thought to be closer to an increase of 28% [5]. The population also appears to have spread with the most recent spatial description suggesting a 25% increase in area occupied within the estate [18]. Information about the distribution or abundance of feral wild boar in the wider landscape are unavailable, as is any quantitative analysis or description of the hunting effort.

### Model framework

#### Overview

The model explicitly simulates the life history of individual boar, referred to as agents, living and moving between contiguous patches in a predefined landscape derived using a randomised Thiessen’s polygon based method. Each unique agent is represented by their spatial location (patch), sex, age, life stage (infant, juvenile or adult), disease status (susceptible, infected-infectious or recovered-immune), and if female by their current reproductive status (time pregnant, current litter etc.). Patches simulate the real landscape of the Forest of Dean at a scale sufficient to host 20–30 boar per patch, each described by individual environmental properties (i.e. those necessary for simulating the population dynamics of feral wild boar, the epizootiology of their diseases, or the geography of management interventions) and identify a list of direct neighbours into which boar can disperse. Key landscape properties include: a continually updated list of the boar inhabitants, an action threshold (computed as the proportion of available woodland multiplied by a typical density of boar in Europe of 10 per km^2^) describing the density at which individuals begin to experience pressure from overcrowding; a carrying capacity describing the density at which population growth should reach zero (computed as the assumed patch quality multiplied by a maximum density of 50 per km^2^, the patch quality is the proportion of available woodland habitat plus half the proportion of grassland for foraging; see [14]). It is important to note that patches here do not necessarily define the home range of an individual or specific sounders (maternal social group) but could in fact contain a number of sounders which interact with each other more frequently than with those of neighbouring patches. Each landscape is therefore a single realisation of how space, and thus contact rates, could exist in the real world. The geography of real world features which may mediate the shape or size of patches as well as contact between them is represented directly in the simulated landscape: in this study canals, rivers more than 10 metres wide and major roads define the edges of patches as they are considered to represent effective barriers to the daily movement of boar, but these features only stop dispersal if the distance between patch edges is more substantial. We assume that distances greater than 200 metres (e.g. across the River Severn) although physically traversable present sufficient challenge such that the probability of crossing is effectively zero.

**Fig 2:**
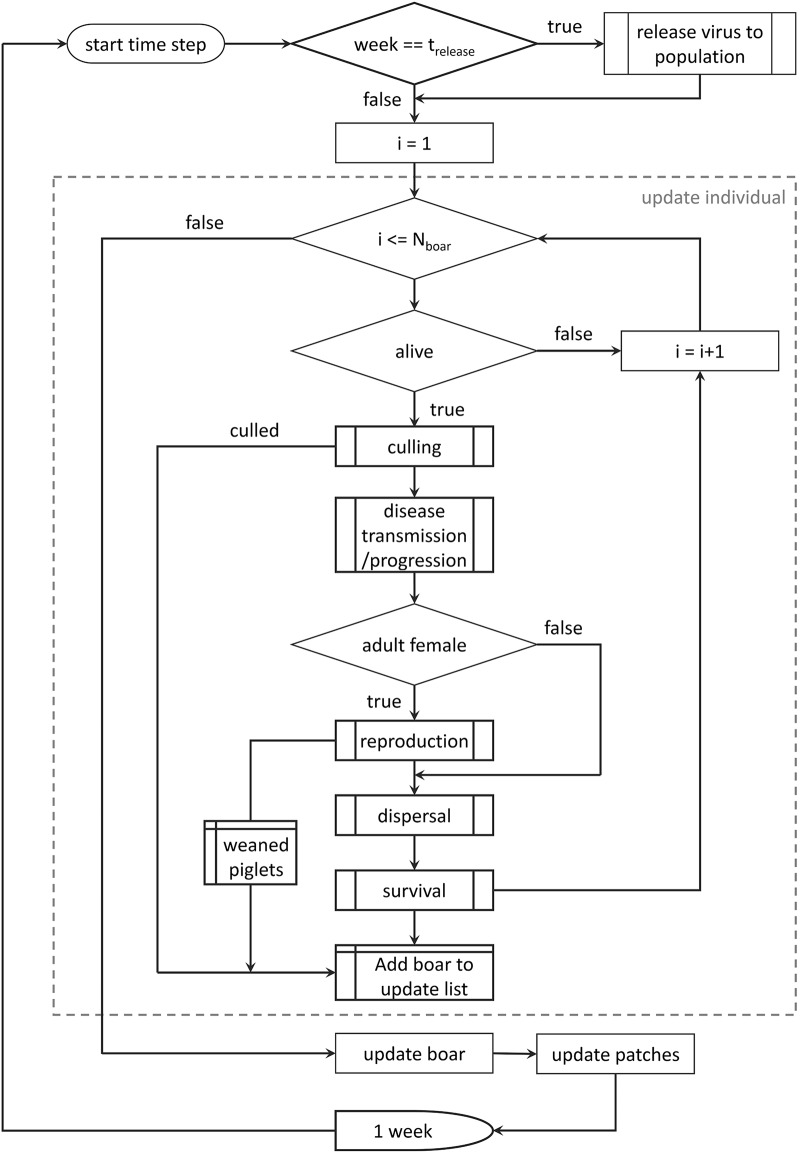
Model diagram. Flow chart outlining the updating process applied in the model.

To represent the rapid dynamics of a highly contagious disease such as FMD the model operates on a weekly time step and the pathology of FMD is so rapid that we consider here that infected animals are also infective and use the terms interchangeably. Individuals are updated in order based on a fixed snapshot of the population from the previous week applying stochastic tests (comparison between a number drawn at random, typically on the range 0 to 1 and a fixed threshold for success) to represent various ecological and environmental processes: reproduction (breeding, gestation, birth and weaning); survival (natural mortality, culling / hunting, old age); dispersal (inter-patch movement); and disease transmission / progression); according to the algorithm illustrated in Fig 2.

#### Reproduction

All adult females are able to breed within a typical season from October to May. Individuals mate and become pregnant according to a seasonal density-dependant probability scaled to account for the ratio of available males to female; males are assumed to be able to mate with a maximum of 10 females per week, similar to domestic pigs. Once pregnant, a counter is used to track the gestation period of sows with their litter size drawn at random from a Poisson distribution with a density-dependant mean. Piglets are initialised as explicit agents and assigned as a property of the mother to simulate dependence whilst weaning. Their sex is assigned at random (see Table 1 for sex ratio) and if all piglets die during weaning then the female begins a period of recovery and might breed again in that year. Once weaned piglets are transferred to the main population as juveniles, and their mothers enter a period of recovery designed to control the proportion of second litters at the end of which they are free to reproduce again if still within the breeding season.

**Table 1:** Population parameter values used in the model.

Parameter	Value	References
Maximum weekly survival probability	0.99 (Infant), 0.993 (Juvenile), 0.996 (Adult)	[6]
Survival reduction factor at carrying capacity	0.976 (Infant), 0.978 (Juvenile), 0.992 (Adult)	
Male fecundity (max. partners per week)	10	
Female fecundity (prob. of successful breeding)	0.026 (Infant), 0.082 (Juvenile), 0.123 (Adult)	[6]
Female fecundity reduction at carrying capacity	0.176 (Infant), 0.306 (Juvenile), 0.276 (Adult)	
Maximum mean litter size	4.5 (Infant), 6.5 (Juvenile), 6.8 (Adult)	[6]
Litter size reduction factor at carrying capacity	0.596 (Infant), 0.472 (Juvenile), 0.866 (Adult)	
Breeding cycle (weeks)	18 (Pregnancy), 12 (Weaning), 6 (Recovery)	
Sex ratio (M:F)	0.5 (1:1)	
Maximum weekly settle probability (not move)	1	
Settling reduction at carrying capacity	0.976 (Infant), 0.978 (Juvenile), 0.992 (Adult)	
Movement kernel (patches)	6 (Male), 4 (Female)	
Life stages (weeks)	3 (Infant), 12 (Juvenile), 104 (Male maturation), 35 (Female maturation), 416 (Female senility), 780 (Maximum age)	
Culling efficacy (proportion per week)	0–0.012	

#### Survival

Boar are removed from the model by one of three processes; natural extrinsic mortality, anthropogenic action (i.e. culling and hunting) and if they survive these, an intrinsic mortality process (old age). Extrinsic mortality is decided by a stochastic test against a density- (relative to patch carrying capacity) and stage- (piglet, juvenile or adult) dependant probability. Individuals failing this test die and the remaining steps in the update are skipped. Piglets are tested as part of the reproduction cycle. However, if the mother of piglets dies, we assume they also die unless there is another female within the patch to provide maternal support.

Culling or hunting occurs at a fixed individual rate, with this described by the location of the patch and the scenario; culling represents the organised and systematic removal of boar from the FC estate whilst hunting represents the *ad-hoc* removal of boar in the wider landscape. Both are described by the individual likelihood of individuals being culled or hunted. A stochastic test is performed to determine whether an individual is removed against their patch’s specified culling / hunting probability. As in the case of natural mortality individuals failing this test are removed from the model immediately.

In this study boar are not allowed to live beyond a maximum age (Table 1) and are removed when this is exceeded. The variable used to track an agent’s age is also used to define their maturation across age classes (suckling piglet > immature juvenile > breeding adult).

#### Dispersal

Daily movement of boar and their individual or sounder home ranges occur within the patch and are not represented directly within the model. The likelihood of individual dispersal to a new patch is based on a density-dependant probability. As the population of a patch approaches its carrying capacity the more likely an individual is to move. If an individual opts to move, a destination is selected at random from neighbouring patches weighted according to the relative length of shared boundary. In order to produce a realistic dispersal kernel a fixed number of moves are permitted per update step performing a probability test after each move. Individuals continue to move until they fail a probability test, are unable to move any further without returning to the same patch or run out of movement steps. For dispersing individuals, the character of the recipient patch is applied immediately to any outstanding update tests in a time-step.

#### Disease transmission/progression

Susceptible individuals are infected with a probability reflecting the sum of two sources of infection (direct transmission from infective animals and indirect transmission from the contaminated environment) in both the local patch and its immediate neighbours. Infection is density-dependant within the same patch and proportionate to the interaction between adjacent patches described in the parameterisation section. The calculation is expressed as follows:

$$p_{i}^{inf}=({{(1-p}^{dir})}^{I_{i}}{{(1-p}^{ind})}^{D_{i}}\prod_{j}^{n}w_{j}{{(1-p}^{dir})}^{I_{j}}{\left(1-p^{ind}\right)}^{D_{j}}),$$

where $p_{i,j}^{inf}$, *I*_*i*,*j*_ and *D*_*i*,*j*_ are the probability of infection, number of infected conspecifics and expected doses of contaminated material likely to be ingested (number of doses present multiplied by an ingestion factor) in patch *i* and *j* respectively, *p*^*dir*^ is the probability of direct infection from one infected conspecific, *p*^*ind*^ is the probability of infection from ingesting one median tissue culture infectious dose (TCID50) of contaminated material and *w*_*j*_ is the contribution of infection from neighbouring patch *j* according to the interaction between patches *i* and *j*.

The length of infection (duration in weeks) is drawn at random from a gamma distribution with parameters as outlined in Table 2; this distribution is defined such that the average period of infection is equivalent to 1.14 week (8 days) [15]. To reflect the physical impact of infection modifiers (otherwise set to 1) are used to influence probabilities of successes within processes such as survival and reproduction. In this case we only consider the strain of FMD to reduce female fertility affecting reproductive capacity. There is no impact on survival (i.e. the disease is non-fatal). Once an agent has recovered from FMD (expired infection) it is considered immune at which point survival and reproduction modifiers are returned to 1; following Lange [15] immunity is considered to be lifelong. All offspring are considered susceptible at birth but maternal immunity can be transferred for a period following recovery; up to the number of weeks defined for maximum antibody persistence. At the point of recovery a counter is initialised and decremented over subsequent time steps to track this protection until it reaches zero, at which point no immunity is passed to offspring. It should be noted that unlike Lange [15] we do not explicitly define a maximum duration of immunity from maternal antibodies, which they state as 12 weeks, as we interpret this to reflect the weaning period. We therefore assume that whilst suckling, piglets remain immune but do not acquire immunity beyond this period (i.e. immunity does not last for up to 12 weeks after suckling ends).

**Table 2:** Epidemiological parameter values used in the model.

Parameter	Value	References
Probability of direct infection (between individuals)	0.25	[29, 30]
Probability of infection from ingestion of one TCID50	0.003	[31]
Ingestion factor	0.000001	[15]
Daily excretion by boar	10^6^ TCID50	[32–34]
Decay curve (A, B ~ Ae^-Bθ^)	17.838, -0.1579	[15, 35]
Neighbour transmission factor (forage overlap in km)	0.4	[15]
Gamma distribution of infectious period (mean, shape)	1.0, 5.0	[29, 36]
Persistence of maternal antibodies	15 weeks	[37]
Infant survival reduction if ill	0.5	[15]
Fertility reduction if ill	0.625	[15]

Patches can harbour a residual “load” of environmental contamination. Disease “load” represents an abstract description of the sum of infective material present in a patch, including that added by new immigrants, deposited by currently infected resident animals or even from brief visits by neighbours, as well as historical components of the disease load contributed in previous weeks which are subjected to a gradual decay process. Following Lange [15], this decay is modelled using an exponential function based on a daily time step according to a temperature dependant half-life [19] using monthly mean temperature. Temperatures for each patch were extracted from 5 km resolution gridded maps provided by the Met Office [20]. The reader should note that in this study we only consider natural transmission between feral boar and ignore other disease hosts in the British landscape such as livestock (pigs, cattle and sheep) and wild deer, as well as anthropogenic pathways e.g. fomites on hunters.

### Parameterisation

The model was initially parameterised using values from existing literature, empirical studies and other models (Table 1). Density dependant relationships were defined by a sigmoidal function [14] using parameter values according to environmental quality [6] (as a proxy for density pressure). As these published values were for a closed population and hence did not include probability of dispersal; we assumed functions follow the same shape as mortality i.e. in any population animals will try to move rather than stay and die. The shape of the relationship was systematically tuned (within the range of published values) to ensure that the asymptotic limit of population growth in each patch matched the defined carrying capacity.

Parameters for the epidemiological components of the model (Table 2) were adopted from Lange [15]. All were applied directly with the exception of the neighbour transmission factor which scaled within-patch transmission to account for a reduced interaction with conspecifics in neighbouring patches. Lange [15] assumes a factor of 0.1 based on an interaction between 2 km resolution cells. We generalise this value to compute the corresponding width of overlap between patches that would yield such a reduction in interaction (frequency of meetings assuming homogeneous mixing within patches) and consequently infection. Using this value we computed probabilities of transmission between neighbouring patches as a function of the proportion of patch area within overlap distance of the boundary, limited to a maximum proportion equal to 1.

### Simulations

Populations were initialised according to a fixed distribution of boar approximating that described in 2015 [18], with a starting population of 1120 individuals distributed across patches according to average density (weighted by area) extracted from this underlying distribution (note that the starting population is marginally inflated from that reported due to the accuracy of extracted survey maps). For simplicity all individuals were initialised as 2 year-old (sexually mature) adults with sex selected at random (sex ratio: Table 1).

In order to initialise our experimental scenarios with realistic and stable populations (i.e. composition and range of age and sex classes) we ran the model for 5 years without culling (preliminary testing showed that demographics stabilise after approximately 2 years) and subsampled the population in each patch to reset starting populations.

### Culling and hunting effort

In this study culling and hunting are functionally identical, though we distinguish them for the purpose of inference and discussion. This rate was determined by using the model to simulate the recent population and spatial dynamics of the boar in the FC estate. Thus, in the absence of disease, we explored the cull effort required to produce the observed changes between 2015 and 2016 [5]. A cull probability of 0.0065 is required to approximate the reported empirical observations; including a 28% population growth and a spatial spread of 23%.

### Scenarios

Our model permits the exploration of many diverse aspects of the population and spatial dynamics of feral wild boar, as well as the establishment and circulation of suid diseases. Here we initially explore three scenarios in the absence of disease to better understand how hunting might influence the dynamics of the wildlife host and then include FMD in our simulations to focus on two specific issues pertinent to disease risk assessment; the potential for feral wild boar in the Forest of Dean to support the endemic circulation of FMD, as well as the time it may take for the disease to burn-out in scenarios where it is not maintained. Both are important when considering the impact of feral wild boar on regional or national livestock industries or planning interventions against disease.

As culling effort and hunting effort will directly affect the dynamics and distribution of boar in the Forest of Dean we consider three contrasting scenarios where these remain fixed across the duration of the simulation (50 years); where hunting effort is identical to culling effort within the FC estate (hunting = 0.0065), where it is completely absent (hunting = 0) or where it is so efficient it immediately accounts for any boar that appear in the wider landscape (hunting = 1). Anecdote suggests that the current hunting effort in the wider landscape is patchily distributed and may be limiting, but possibly not preventing the spread of boar.

As simulations of FMD are sensitive to the abundance and distribution of boar at the moment of focal infection, we address this by initiating disease at varying time-points within each 50 year scenario (0, 3, 5, 10, 20, 30 and 40 years). For each simulation disease was introduced to a single boar at random (thus also into a randomised location) resulting in simulations testing the epizootiology of FMD in small restricted populations early in each prediction, whilst in others disease may be introduced in to an expansive and abundant population at more distant future dates. Simulations of all three contrasting hunting scenarios were repeated with the introduction of FMD permitting a prediction of the interaction between management and disease. The interval between the introduction of FMD and the eventual disappearance of the last infected individual was taken as the time taken for the disease to be eliminated (i.e. disease eradication).

Ten randomised instances of each scenario were run across 10 randomised simulations of our study area, producing 100 unique repetitions from which quantitative probabilistic descriptions of our scenario outcomes were produced. The resulting outputs described, for each patch, the number of boar in each demographic class as well as the number culled, died naturally, breeding, susceptible, infected and immune are combined with the specific landscape map and aggregated to produce the average, or predicted, population response. Specifically this includes; change in total population, change in area occupied and, from initial release, the time to disease freedom.

## Results

**Fig 3:**
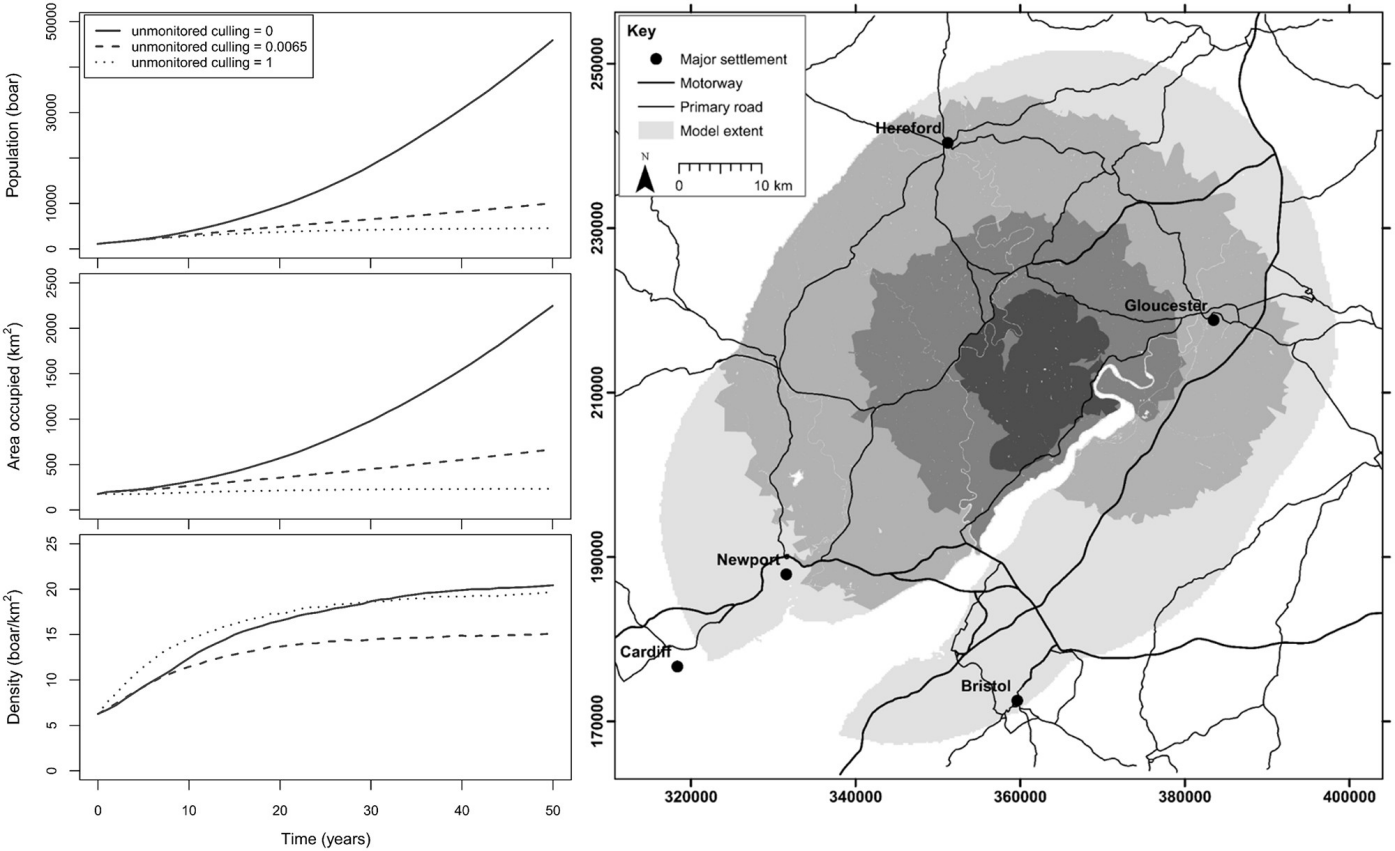
Population growth under various management scenarios. Time series plots (left) show: total population (top; L_top_); total area occupied (middle; L_mid_); mean density (bottom; L_btm_); at fixed recording week, corresponding to the timing of the annual surveys conducted in the Forest of Dean, each year of the 50 year simulation period for various levels of hunting (unmonitored culling) on the private land beyond the FC estate. Map (right) shows the maximum extent of populations (boar present in at least one simulation) after 50 years of simulation under each of these management scenarios. Darker shades of grey denote increasing levels of unlicensed hunting on the area outside of the FC estate, where culling can be regulated, with the darkest shade reflecting immediate removal (unmonitored culling = 1) and the lightest (excluding region denoting model extent) reflecting no control (unmonitored culling = 0).

Simulations predicting the abundance and distribution of feral wild boar in the absence of FMD show that in the most conservative scenario where hunting results in the immediate removal of animals across the wider landscape (Hunting = 1) the population in the core area rises rapidly to an asymptotic limit approaching the assumed mean carrying capacity of the FC estate; approximately 4,000 individuals with average densities of 20/km^2^ (Fig 3; L_top_). The area occupied remains constant at around 200 km^2^ (Fig 3; L_mid_). In the intermediate case, where hunting effort is equal to the culling effort (i.e. uniform removal rate across the entire landscape), the results suggest that boar spread into the wider landscape (beyond their current distribution) and consequently we observe a steady rise in both total numbers and the area they occupy. Without disease, simulated populations increase to a mean of nearly 9,000 individuals spread across approximately 600 km^2^ after 50 years; these rapidly reach and then maintain an average density of approximately 15 individuals/km^2^ indicating matching rates of increase in numbers and area occupied. Where hunting pressure is absent across the wider landscape, simulations predict that populations grow rapidly to reach a mean of 50,000 individuals after 50 years at equilibrium densities close to the average carrying capacity of patches, around 20 individuals/km^2^. This management scenario shows that if left unchecked, boar populations could reach or exceed our study extent within this relatively short period. In all scenarios without disease population density rapidly increases to equilibrium after 10–15 years at which point the rates of population increase and expansion can be considered identical (comparing the plots L_top_ and L_mid_ of Fig 3) i.e. greater abundance necessarily reflects a larger area occupied.

**Fig 4:**
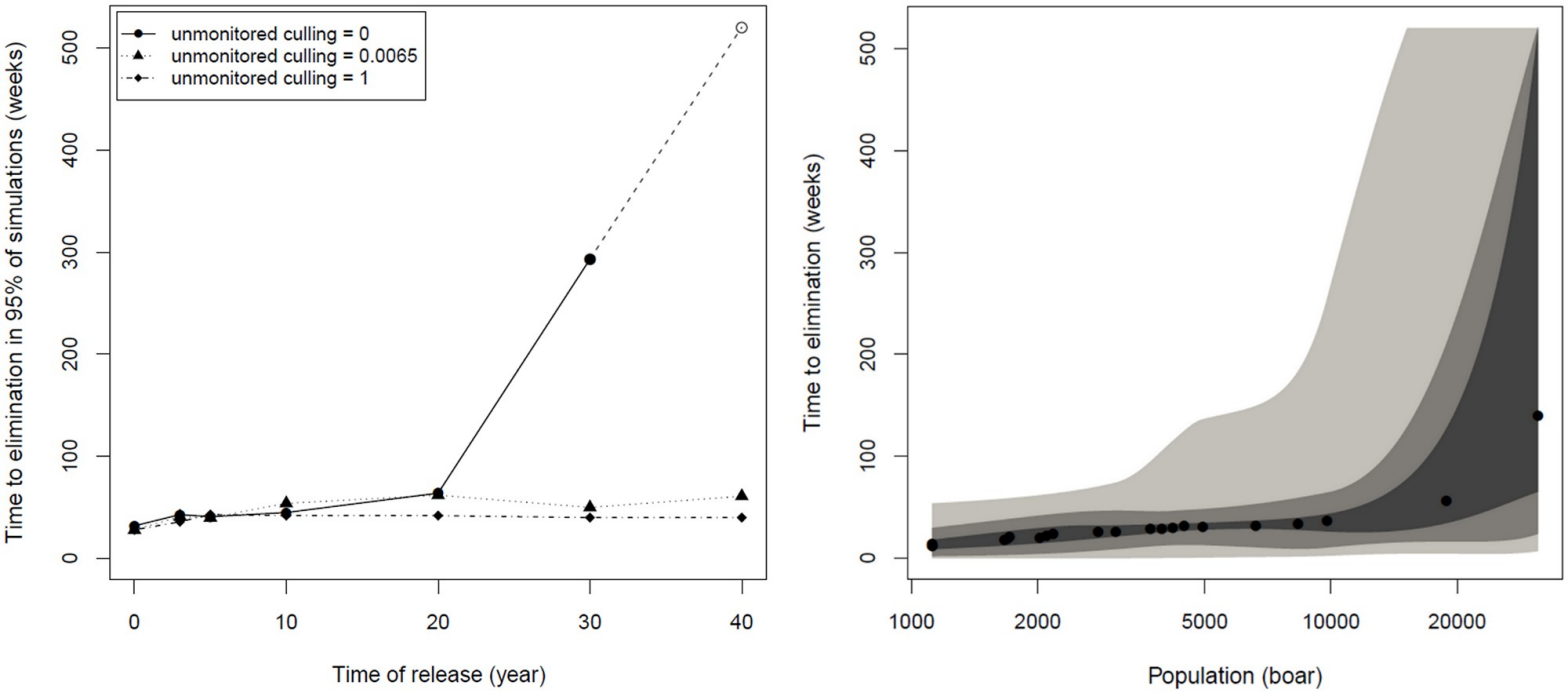
Time to elimination following an outbreak. (Left) Plot shows the time to reach zero infected animals in 95% of model repetitions against year in which a single individual infected with FMD is released into the population for a range of potential management scenarios exploring the impact of unknown culling effort on land beyond the current distribution on publically owned land; culling levels tested simulate no culling (= 0), equal to FC land (= 0.0065) and complete removal (= 1). (Right) Plot shows the median time to reach zero infected animals (dots) against population size at time of release taken across all management scenarios. Shaded regions denote smoothed ranges centred on the median containing (from darkest to lightest): 50%, 90%, and 100% of model repetitions.

Predictive simulations of the epizootiology of FMD in feral wild boar across the Forest of Dean suggest that the disease will usually burn through the population, becoming self-limiting (Fig 4). Alternative management scenarios did change the time to disease freedom, with those simulations including substantial hunting effort in the wider landscape usually indicated the disappearance of FMD within one year (30–50 weeks) regardless of the timing of release and thereby population size (although the range of population sizes represented by these simulations is relatively narrow). However, where populations are allowed to grow (intermediate hunting effort across the wider landscape) the time to disease elimination can become prolonged. In the absence of hunting, disease introduction after 20 years where populations exceed 10,000 individuals across 500 km^2^ resulted in FMD persisting for up to 10 years in some simulations (<5%). FMD persistence became more likely as population size increased further up towards 25,000 individuals across 1,250 km^2^ where the likelihood of infection persisting for 10 years approached 95%.

## Discussion

Here we describe a spatially explicit individual-based population model to simulate the growth and spread of an introduced population of feral wild boar in the Forest of Dean and surrounding area. This model was designed to both facilitate realistic scenario formulation and minimise biases in its expression of its underlying landscape [21]. The model was also designed to reproduce the population dynamics and spatial dynamics of feral wild boar across a real-landscape as well as the epizootiology of FMD within this wildlife host. Using this model we explored the effect that culling within the FC estate and hunting across the wider landscape have on the expansion of this population as well as the likelihood of a prolonged outbreak of FMD in feral wild boar.

The combination of our representation of a real-world landscape, along with an individual-based wild boar population model appears to simulate feral wild boar biology sufficiently well to simultaneously produce population growth and spread similar to that observed recently in the Forest of Dean. Formal validation of our spatially explicit model of boar in this landscape is limited by the sparse and uncertain empirical data describing this system. Any empirical validation of the epizootilogical component of the model (FMD) is currently impossible and its representation in this study is necessarily theoretical. More detailed accurate information on the current distribution and of the actual number of feral wild boar taken by hunters is also needed to increase the reliability of our predictions. In particular, the model predicted growth over the first few years which appears inflated as the initial high density population spreads out; in reality populations tend to have lower density at the range edge, resulting in a more stable rate of spread and assuming no resistance from hunting pressure. Recent methods for estimating density of feral wild boar from camera trapping and/or through distance sampling could be used [22, 23] together with engagement of the local land owners to gather this information.

Our simulations suggest that, following an outbreak of FMD in a population similar to that estimated for 2016, the disease would not be sustained in 95% of the simulations, burning out within a year without any specific additional intervention (culling across the FC estate does continue in this scenario). However, the same scenario played out at a more distant future date, when the population is more abundant and widely distributed show that feral wild boar may become a self-sustaining reservoir of FMD, especially in those cases where hunting across the wider landscape is absent. Our results also suggest that population sizes above 10,000 and spread across more than 500 km^2^ have an increased probability of FMD being sustained for substantial periods (at least 10 years) with this risk rising further with increasing numbers of animals.

Our spatially explicit study of the epizootiology of FMD in a wild host suggests an interesting relationship between the persistence of FMD in a population of feral wild boar and its geographical area of occupancy. As a habitat generalist, wild boar find many different ways of living and breeding in many contrasting habitats and whilst the value of local patches, and the densities of boar they might maintain can vary across landscapes, it would be unusual in the UK to find extensive areas uninhabitable by boar. Our study area, across an extent around the Forest of Dean appears relatively benign to boar. Some patches (such as those in the FC estate) are probably of high value to boar, but even those dominated by pasture or mixed arable land-uses will probably include woodlots and scrub-cover and support notable densities in the absence of hunting. Thus as the patches across this real-world landscape appear to be relatively homogenous when describing their value to wild boar, we suggest that the consequential equilibrium densities of boar should also be relatively homogenous. It is unsurprising then that we find a close positive relationship between the absolute abundance of a discrete population and the area of its occupancy. Disease-free simulations here show such a relationship (comparisons of plots L_mid_ and L_btm_ from Fig 3) and to corroborate our interpretation of a generally benign and homogenous environment we note that patch extinction after establishment is quite unlikely, the rate of population spread is relatively consistent and populations usually spread to occupy single contiguous areas. Thus despite our model’s ability to reflect the heterogeneity of real-world landscapes and variation in real-world host densities, both in this study appear relatively homogenous across our simulations. In this context, the variation in the epizootiology of FMD (especially the likelihood of becoming endemic as well as the duration of an outbreak) appear most directly related to the geographical description of population, and not its aspatial abundance. Here endemic FMD appears to occur when populations become so geographically extensive that the movement of disease (here specified by weekly patch to patch transmission rates) interacts with the breeding cycles of boar such that new susceptible animals are produced before every existing animal is infected. For highly contagious diseases such as FMD the absolute number of boar in each patch (or their density) and the absolute abundance of the whole population is less important as we assume that most of those within a patch have frequent and regular contact with each other and rapidly become infected, and the change in rate of transmission across progressively larger distances is key in predicting the duration of an outbreak.

Work here suggests the value that proactive and organised, or prophylactic, management of feral wild boar might have in mitigating the risks or impacts produced by an outbreak of FMD in this region. Simulations where efficiently distributed hunting effort is deployed to manage feral wild boar appear to help restrict their expansion and may sufficiently limit population growth to reduce the duration of an outbreak. However, our scenarios make two assumptions that require comment. The first is that our description of hunting effort represents an idealised and regionally co-ordinated management plan. In this study boar are removed in perfect proportion to their density. In reality, hunting effort across a landscape composed of a mosaic of privately owned properties of varying size will be far less efficient, because some landowners may not permit hunting, others may waste effort in patches of low density (either because they are most sensitive to the impact of boar or because they cannot gain access to more densely populated patches), whilst some landowners hosting patches most favourable to boar may be happy to maintain relatively dense populations. Secondly, we assume no further releases of boar away from the core area in the Forest of Dean. Where newly established foundling populations remain geographically distinct, the risk of endemic FMD depends mainly on the area occupied by the single largest contiguous population. As previously dis-contiguous populations merge, our earlier observation suggest that the risk of endemic FMD, or the duration of impacts following an outbreak may increase disproportionately in this landscape. Thus one theoretical strategy to lower such disease risks might be to deliberately fragment extensive contiguous populations using targeted culls [24].

We suggest that the rate of culling we calculate from the existing evidence across the FC estate [5] is realistic (removal rate of approximately 0.65% of the population per week) but is insufficient to maintain the current population of feral wild boar within the FC estate in the Forest of Dean. We predict that over 50 years this effort will permit the population to grow more than 4 fold within the FC estate. Ineffective, ad-hoc and uncoordinated hunting pressure across the wider landscape may produce substantial population growth; here we calculate this to be 12.5 fold increase in both abundance and the area occupied over the same period (50,000 individuals across 2,500 km^2^ as opposed to 4,000 across 200 km^2^). Where the effort of hunters in the surrounding landscape equals the culling effort within the forest estate, we suggest that population growth and spread will slow and that densities may be held consistently below their patch carrying capacities. However it appears impractical to attempt to maintain densities of boar low enough to inhibit the establishment of FMD. In all our simulations a single infected individual caused an outbreak (secondary infection) even with average densities as low as 5 individuals/km^2^. In addition initial simulations suggest that control of the current population (i.e. prevent population growth and spread) requires a culling / hunting effort at least 1.5 times greater than the current estimate which would have to be applied consistently across our study landscape (i.e. co-ordinated adaptive management in response to the local density). Where such co-ordinated action might require the co-operation of landowners hostile to hunting or in areas where the use of guns is not possible (e.g. towns and villages) it might be possible to consider non-lethal alternatives such as fertility control [25–27] as components in an integrated management strategy implemented over a long term (e.g. UK government 25 year environment plan [28]).

The limitations in available data notwithstanding, the model outputs described here provide an important first step towards generating the evidence required to support robust policy development and decision-making regarding the management of isolated feral wild boar populations. If allowed to expand, unopposed boar could rapidly become a potential wildlife reservoir for FMD capable of sustaining the disease for many years, delaying a return to a disease-free status, and producing serious socio-economic impacts.
